# Chiral recognition of macromolecules with cyclodextrins: pH- and thermosensitive copolymers from *N*-isopropylacrylamide and *N*-acryloyl-D/L-phenylalanine and their inclusion complexes with cyclodextrins

**DOI:** 10.3762/bjoc.7.27

**Published:** 2011-02-14

**Authors:** Sabrina Gingter, Ella Bezdushna, Helmut Ritter

**Affiliations:** 1 Institute of Organic Chemistry and Macromolecular Chemistry, Heinrich-Heine-University Düsseldorf, Universitätsstraße 1, D-40225 Düsseldorf, Germany

**Keywords:** amino acid, chiral recognition, cyclodextrins, LCST, stimuli-responsive polymers

## Abstract

In the present work we report the enantioselective recognition of water soluble stimuli-responsive polymers bearing phenylalanine moieties via host-guest interaction with β-cyclodextrin and randomly-methylated-β-cyclodextrin (RAMEB-CD). We synthesised *N*-acryloyl-D/L-phenylalanine monomers (**2_D_**, **2_L_**) which were then copolymerised under free radical conditions with *N*-isopropylacrylamide (NIPAAm). The resulting copolymers **3_D_** and **3_L_** exhibit a lower critical solution temperature (LCST) of 25 °C. As a further benefit, the presence of a free carboxylic group in the copolymer system gives a high sensitivity to the pH value in respect to the LCST value. The enantioselective recognition of the side groups of copolymers **3_D_** and **3_L_** and their solubility behaviour were investigated by dynamic light scattering and 2D NMR spectroscopy, respectively.

## Introduction

Chiral recognition has attracted much interest not only as a separation technique but also in the pharmaceutical industry where the supply of pure enantiomers for drug purposes is of great importance [1]. Thus, it is inherently important to find reliable recognition systems for chirality in various kinds of molecules. To date only a few papers exist dealing with recognition of chiral polymeric systems by use of cyclodextrins. One of the first published methods was the enantioselective inclusion of isotactic polylactides with cyclodextrin (CD) rings [2].

Cyclodextrins (CDs) are cyclic oligosaccharides comprising six, seven or eight α-1,4 linked chiral glucopyranose units. They easily include suitable molecules with hydrophobic segments [3–6]. Due to their asymmetric centres, CDs have the ability to discriminate between suitable enantiomers. They form diastereomeric complexes with slightly different stabilities [5–10]. Nevertheless, these small energy differences are usually sufficient to achieve an adequate chromatographic resolution [11].

These small energy differences can even be detected by thermal titration or spectroscopic methods, which have been achieved, e.g., for natural amino acids or their *N*-protected derivatives [12–14]. In this connection we have recently shown that in CD-complexed racemic mixture of *N*-methylacrylamidophenylalanine, only the D-enantiomer is preferably polymerised, whereas the L-enantiomer accumulates in the solution [15].

We also showed recently a chiral recognition of polymer attached tryptophan with CD [16]. In this paper we now wish to report the chiral recognition of polymeric attached phenylalanine (Phe) with randomly-methylated-β-CD (RAMEB-CD) and native β-CD.

## Results and Discussion

**Scheme 1:**
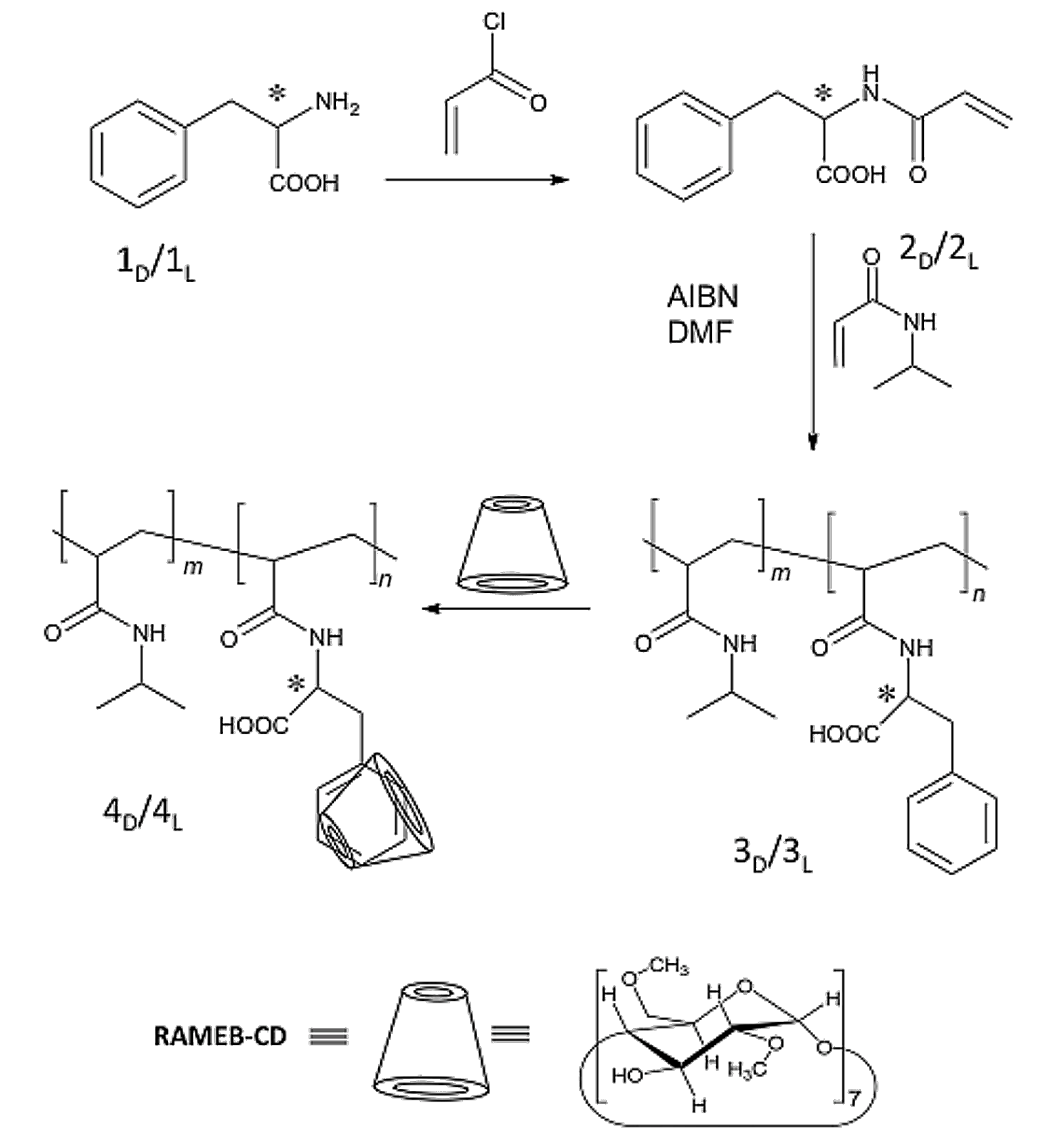
Synthesis of copolymers **3_D_**, **3_L_** and CD-complexes **4_D_**, **4_L_**.

Copolymers **3_D_** and **3_L_** comprising *N*-isopropylacrylamide (NIPAAm), *N*-acryloyl-D-phenylalanine (**2_D_**) and *N*-acryloyl-*L*-phenylalanine (**2_L_**) were copolymerised under free radical conditions with NIPAAm in a molar ratio of 1:20 and 1:10. Compounds **2_D_** or **2_L_** were obtained under Schotten–Baumann conditions using acryloyl chloride. Both copolymers were prepared under similar conditions (Scheme 1). The molar weight distribution of polymers **3_D_** and **3_L_** were determined by gel permeation chromatography (GPC) after silylation of the free carboxylic groups with trimethylchlorosilane.

The resulting copolymer **3_D_**, **3_L_** (1:20) is soluble in water below the critical solution temperature (LCST). However, the presence of D- or L-Phe significantly affects the LCST value of pure poly(NIPAAm) (32 °C), since the hydrophilic/hydrophobic balance of the polymer is changed [17–18]. As noted above, incorporation of the hydrophobic phenylalanine moieties into the polymer reduces the LCST to 25 °C for the **3_D_**, **3_L_** (1:20) copolymer, whereas the **3_D_**, **3_L_** (1:10) copolymer is relatively hydrophobic and not water-soluble. Surprisingly, the polymers **3_D_**, **3_L_** (1:10) become water-soluble due to host–guest interaction with RAMEB-CD yielding **4_D_** and **4_L_**.

**Figure 1:**
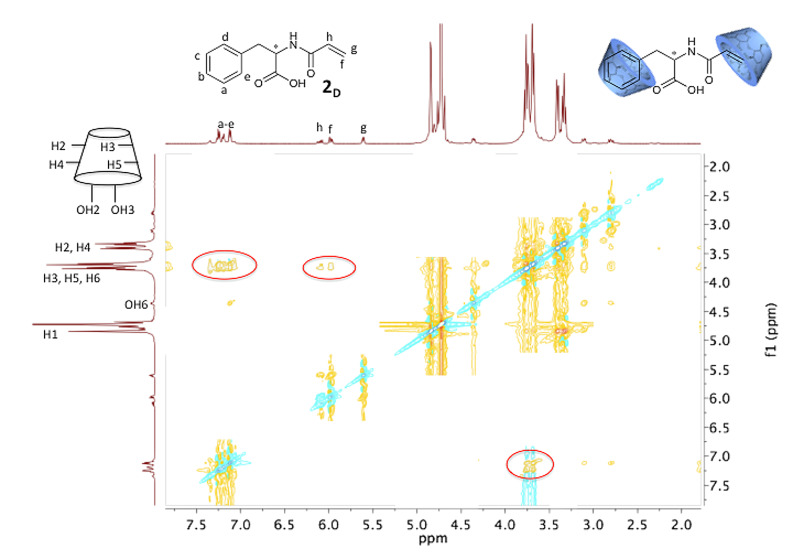
2D NMR ROESY spectrum of monomer **2_D_** with RAMEB-CD.

To confirm the formation of the proposed inclusion complexes between the copolymers and CD, which was added in excess, the corresponding monomers **2_D_** and **2_L_** were used as models to prove complexation. Thus, 2D ROESY NMR spectroscopy was performed to show the correlation between the protons of the β-CD and the protons of the guest **2_D_**. Figure 1 indicates as an example the case of **2_D_** where interactions of protons from the phenyl group with protons of the CD cavity are shown, while no interactions with protons of the outer rim are apparent. Furthermore, there is a second interaction of the protons of the double bond of **2_D_** with the CD cavity. Therefore, it can be concluded, that the complex composition with **2_D_** or **2_L_** has a stoichiometry of 1:2.

**Figure 2:**
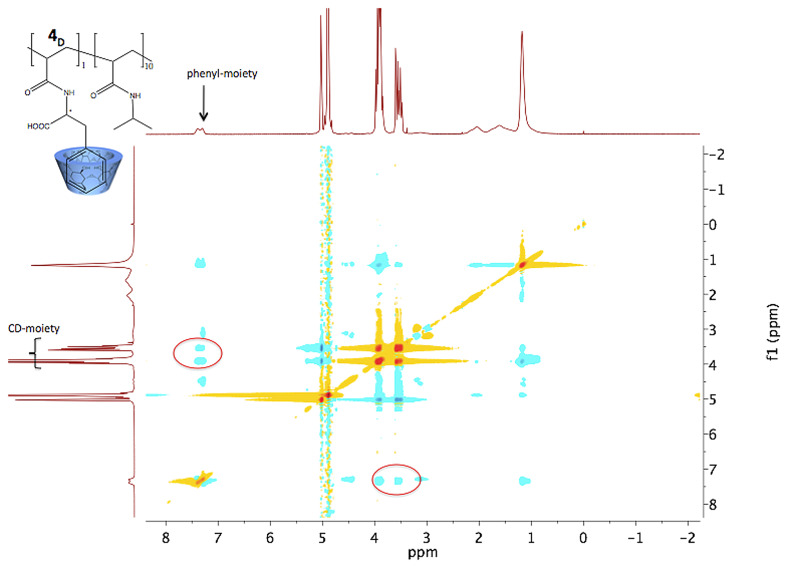
2D NMR ROESY experiment showing the correlation between protons of the phenyl moiety of **4_D_** with the β-CD protons.

To assure for further investigations that the complexation also takes place in the copolymer, additional ROESY experiments were carried out (Figure 2).

The NMR experiments clearly show that the complexation takes place in monomer and polymer solutions, for both enantiomers (also see Supporting Information File 1).

**Figure 3:**
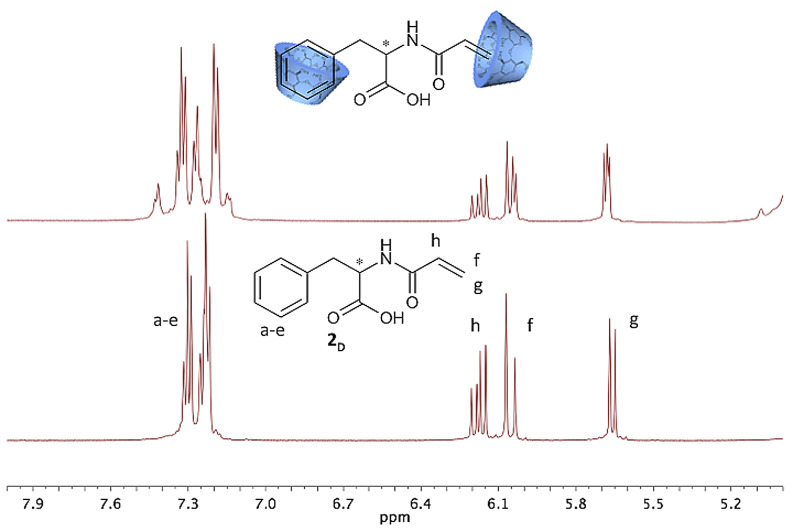
NMR shifts of the complexed monomer **2_D_**.

Furthermore, both a shift of proton signals and the duplication of proton signals can be detected when the spectra of the complex is compared to the pure compounds **2_D_** and **2_L_** (Figure 3).

**Figure 4:**
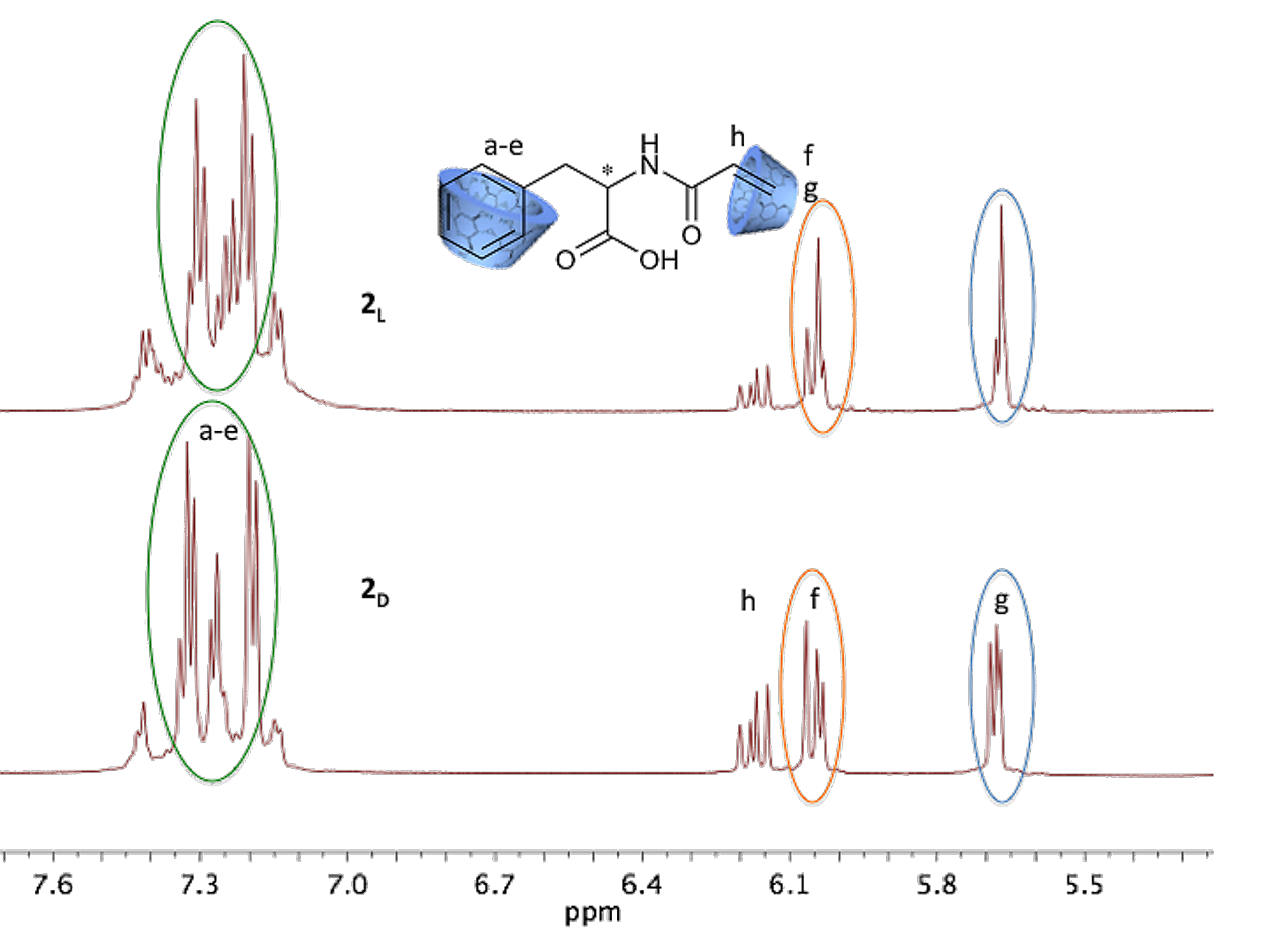
Comparison of ^1^H NMR spectra of **2_D_** and **2_L_** complexed with β-CD.

Figure 3 shows the specific shifts of signals, which occur on complexation. CDs being chiral molecules themselves effect a downfield shift of 0.1 ppm of the aromatic proton signal since the protons are magnetically shielded by the CD cavity. Additionally, each of the aromatic and double bond proton signals, respectively, change from singlets to doublets on complexation. Obviously, the supramolecular structure changes and former magnetically identical protons become non-equivalent protons and appear as doublets. This can easily be observed in case of aromatic protons at 7.31–7.14 ppm and the proton signal of the double bond at 5.6 ppm and 6.0 ppm, respectively. The signal of the proton next to the double bond (6.3 ppm) remains unffected. The described shift of proton signals can be detected for both enantiomers, but with different intensities as can be observed in Figure 4.

As expected, we found that ^1^H NMR spectra of complexed compounds **2_D_** and **2_L_** with β-CD show differences in the splitting pattern of the proton signals (Figure 4). Therefore we conclude that the complex stability is different for **2_D_** and **2_L_** and **4_D_** and **4_L_**, respectively.

**Figure 5:**
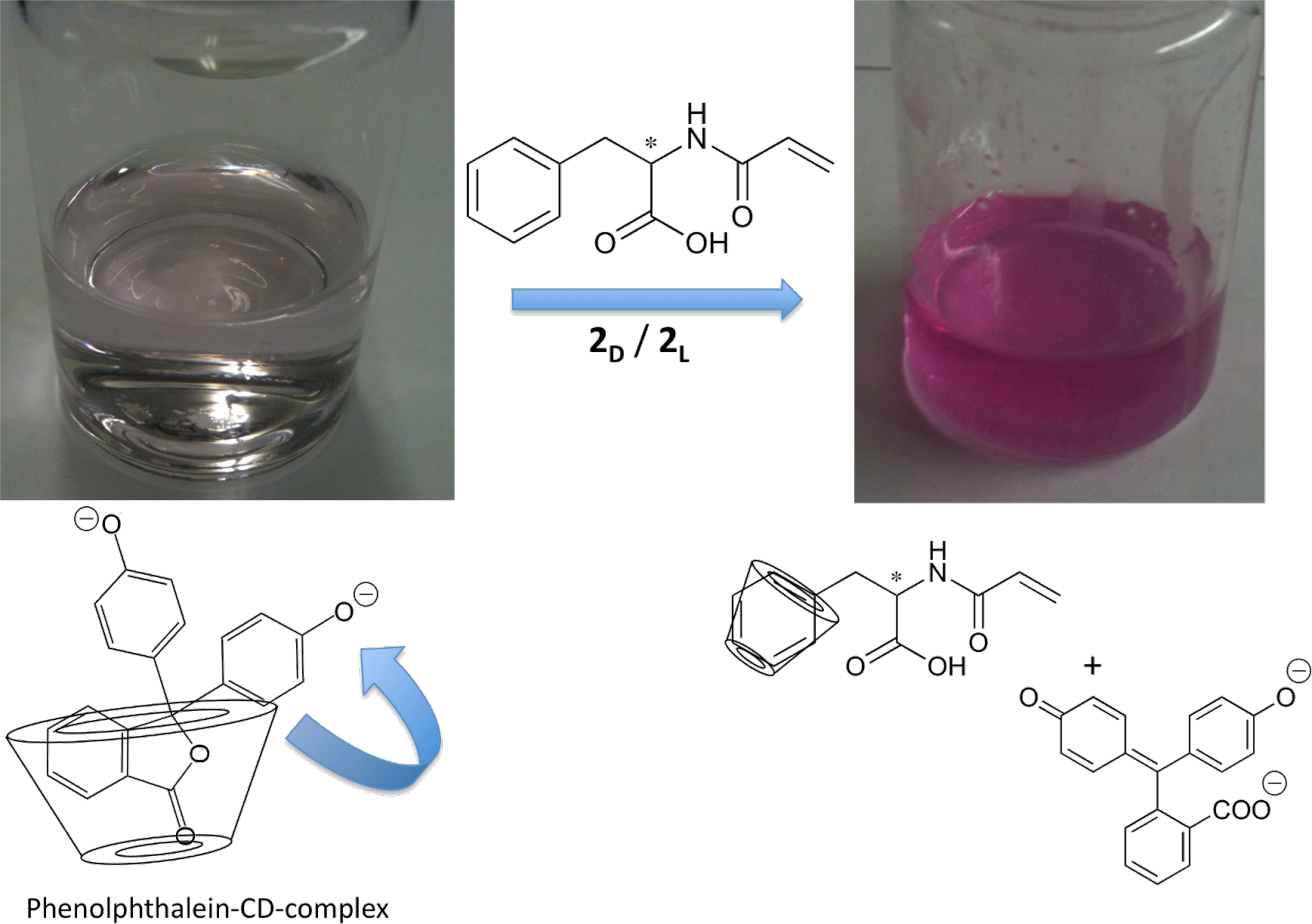
Complex formation with phenolphthalein and phenylalanine as competitor.

To further prove the complexation process of β-CD as host and the Phe derivatives **2_D_** and **2_L_** as guests, we visualized the complexation utilising phenolphthalein [19], i.e., the complexation and decomplexation of a dye with β-CD in basic medium can be employed as a method to prove these processes [20]. In basic media, phenolphthalein exhibits its characteristic pink colour, caused by its planar configuration and electron delocalisation. On adding cyclodextrin to the solution, phenolphthalein becomes colourless in the complexed state. Hydrogen bond formation is accompanied with a conformational change as the planar state of phenolphthalein is reversed which leads to loss of colour. Taking this into account, the addition of a competitive guest molecule should displace the included phenolphthalein and turn the colour of the solution from colourless to pink. Accordingly, the addition of **2_D_** followed by stirring for one hour caused the solution to regain its characteristic pink colour as Figure 5 shows.

**Figure 6:**
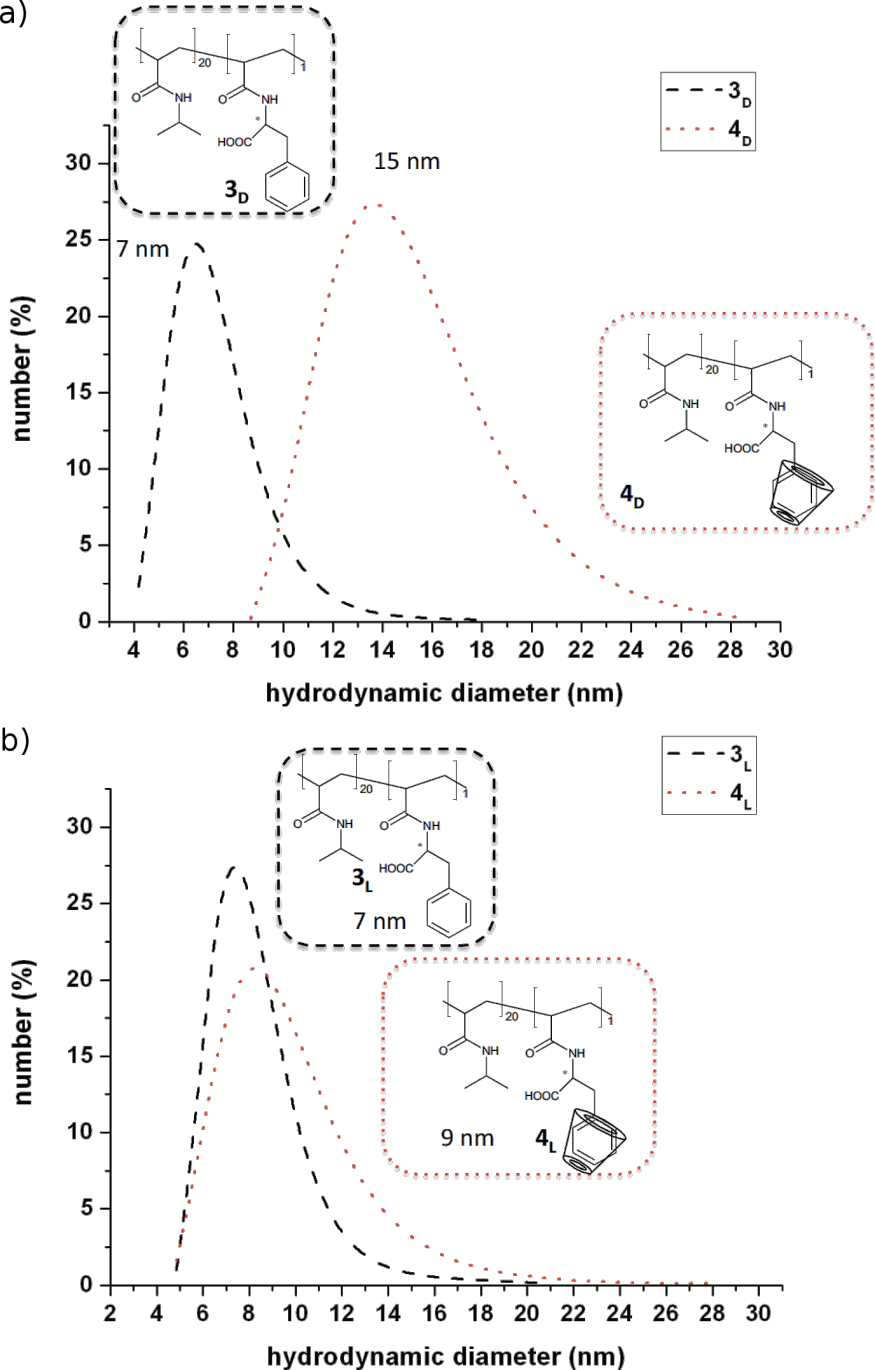
Hydrodynamic diameters of the copolymers and their corresponding complexes with RAMEB-CD a) L-phenylalanine containing copolymer b) D-phenylalanine containing copolymer (1 mg/mL, pH 4).

To prove further the influence of complexation of chiral polymers **3_D_** and **3_L_** with chiral RAMEB-CD, we employed dynamic light scattering to measure particle size and hydrodynamic diameters of the supramolecular structures (Figure 6).

Evidently, the **3_D_** and **3_L_** copolymers possess nearly identical mean coil sizes as expected from the fact that the synthesis of both polymers was carried out under nearly identical conditions. However, the chiral RAMEB-CD causes hydrodynamic diameters which are affected in dramatically different ways for the polymer attached enantiomers.

## Conclusion

In this work, we have succeeded in showing enantioselective recognition of copolymers containing D- or L-phenylalanine moieties by use of DLS and NMR spectroscopy. The results clearly indicate that enantioselective recognition of the polymeric attached chiral amino acid takes place due to host–guest interaction with CD. The *D*-enatiomer of polymeric attached Phe shows a larger increase in hydrodynamic diameter than the L-enantiomer on CD-complexation. In addition, differences in the splitting pattern of ^1^H NMR spectra of CD-complexed monomers were detected. The complexed **2_D_** exhibits a distinctive splitting pattern compared to **2_L_**. We also demonstrated that this complexation of phenylalanine containing polymer can be visualised by the use of phenolphthalein.

## Experimental

### Materials

All reagents used were commercially available (Aldrich Co, Acros Organics) and were used without further purification. Randomly methylated β-cyclodextrin (RAMEB-CD) and β-cyclodextrin were obtained from Wacker Chemie GmbH, Burghausen, Germany, and were used after drying overnight under vacuum (oil pump) in the presence of P_4_O_10_. D- and L-phenylalanine (98.5%) were purchased from Alfa Aesar GmbH & CoKG, Germany. Acryloyl chloride (97%) and *N*-isopropylacrylamide (NIPAAm, 97%) were obtained from Aldrich, Germany, and used as received. Azobisisobutyronitrile (AIBN) (96%) and *N,N*-dimethylformamide (DMF) were purchased from Fluka, Germany. Dimethylsulfoxide-*d*_6_ (99.9 atom % D) and deuterium oxide D_2_O were obtained from Deutero GmbH, Germany.

### Measurements

^1^H NMR was performed using a Bruker Avance DRX 500 spectrometer operating at 200.13 MHz or 500 MHz for protons in DMSO-*d*_6_ or deuterium oxide (99.9%) as solvents. The δ scale relative to tetramethylsilane was calibrated to the solvent value δ = 4.79 ppm for D_2_O and 2.51 ppm for DMSO-*d*_6_. Copolymer compositions were determined by ^1^H NMR.

Infrared (IR) spectra were recorded on a Nicolet 6700 FT-IR spectrometer equipped with a diamond single bounce ATR accessory at room temperature. Turbidity measurements were determined using a TP1 turbidity photometer over a temperature range of 15 to 70 °C. During continuous stirring, the transparency of the sample was determined by a voltage controlled semiconductor laser and a silicium-photodiode at a wavelength of 500 nm and a heating or cooling rate of 1 °C·min^−1^. All critical temperatures were detected by determination of the temperature where the transparency of the solution was 50% of the initial value. The hydrodynamic diameters of the copolymers were determined by dynamic light scattering (DLS) in backscattering mode on a Malvern Zetasizer Nano ZS ZEN 3600 with a laser wavelength of 633 nm and a detection angle of 173°. Measured solutions contained 1 mg/mL substance in water and were performed in a polystyrene cuvette with a layer thickness of 1 cm. Measurement results are calculated by the non-negative least-squares (NNLS) algorithm. Depending on measurement, number- or volume-averaged diameters are used for characterization. Each experiment was performed at least five times. Molecular weights and molecular weight distributions were measured by size exclusion chromatography (SEC) with a Viscotek GPCmax VE2001 system that contained a column set with one Viscotek TSK guard column HHR-H 6.0 mm (ID) × 4 cm (L) and two Viscotek TSK GMHHR-M 7.8 mm (ID) × 30 cm (L) columns at 60 °C. *N,N*-Dimethylformamide (DMF, 0.1 M LiCl) was used as eluent at a ﬂow rate of 1 mL·min^−1^. A Viscotek VE 3500 RI detector and a Viscotek Viscometer model 250 were used. The system was calibrated with polystyrene standards with a molecular range from 580 D to 1,186 kD.

Polarimetry measurements were performed at *T* = 20 °C in 0.1 N sodium hydroxide solution (λ = 590 nm).

#### Synthesis of D/L-acryloylphenylalanine 2_D_, 2_L_

3 g (18 mmol) D/L-phenylalanine were dissolved in 30 mL of sodium hydroxide solution 1.44 g (3.6 mmol) and 1.8 mL (18 mmol) of acryloyl chloride were added dropwise with stirring at 0 °C. The mixture was stirred for 3 h at 0 °C and then at 25 °C overnight. The product was then precipitated. To complete the precipitation, the solution was acidified with 100 mL of ice cold 1 N HCl, the solid separated by filtration, washed with water and lyophilised.

Yield: 50%, FT-IR (diamond cm^−1^): ν = 3341(NH amide), 1709 (C=O), 1648 (C=O amide I), 1594 (Ar), 1532 (NH amide II), 1495, 1455, 1437, 1251, 1222, 1112, 1065, 990, 914, ^1^H NMR (200 MHz, DMSO-*d*_6_) δ (ppm) 8.47 (s, 1H), 7.31–7.14 (m, 5H), 6.30 (dd, *J* = 10.0, 17.1 Hz, 1H), 6.06 (dd, *J* = 2.4 Hz, 17.1 Hz, 1H), 5.60 (dd, *J* = 2.4 Hz, 9.9 Hz, 1H), 4.53 (*J* = 4.9 Hz, 9.5 Hz, 1H), 3.12 (dd, *J* = 4.9 Hz, 13.8 Hz, 1H), 2.91 (dd, *J* = 9.6 Hz, 13.8 Hz, 1H), Polarimetry: λ = 590 nm, *T* = 25 °C, **2_D_** [α]^D^_20_ = −53, **2_L_** [α]^D^_20_ = 53.

Elemental analysis: theoretical C 65.7%, N 6.4%, H 6%, found **2_D_** C 65.8%, N 7%, H 6.5%, **2_L_** C 64.2%, N 5.7%, H 6%.

#### Synthesis of poly(NIPAAm-*co*-D- or L-phenylalaninacrylamide) 3_D_, 3_L_

6.2 g (54 mmol) of *N*-isopropylacrylamide (NIPAAm) were dissolved in 5 mL of dimethylformamide. In each of two 100 mL flasks was placed 0.3 g (1.4 mmol) **2_D_**/**2_L_** dissolved in 3 mL of DMF. Then 68 mg (1 wt %) of azobisisobutyronitrile (AIBN) was dissolved in 2 mL of DMF and the two solutions of NIPAAm and AIBN were divided equally and added to the two flasks containing the monomer solution. The flasks were then flushed with nitrogen for at least 15 min. Both flasks were heated in the same oil bath for 23 h at a constant temperature of 80 °C, the reactions stopped and the products obtained by addition of diethyl ether and washed with diethyl ether. The products were purified by dialysis in a micropore 1000. Yield: 70%, ^1^H NMR (500 MHz, DMSO-*d*_6_) δ (ppm) 8.06–6.49 (m, 1H), 3.84 (s, 10H), 2.14 (dd, *J* = 365.4 Hz, 367.1 Hz, 9H) FT-IR (diamond cm^−1^): ν = 3288 (NH amide I), 2970, 2932, 1713 (C=O), 1641 (NH amide II), 1538 (Ar), 1457, 1386, 1256, 1172, 1095, SEC (DMF): *M*_n_ (**3_L_**) 16000 g/mol; PDI (**3_L_**) 3.6, *M*_n_ (**3_D_**) 7000 g/mol, PDI (**3_D_**) 2.

## Supporting Information

File 1 Additional 2D NMR ROESY and ^1^H NMR data.
