# Supplementary material for: Chiral recognition of macromolecules with cyclodextrins: pH- and thermosensitive copolymers from N-isopropylacrylamide and N-acryloyl-D/L-phenylalanine and their inclusion complexes with cyclodextrins
Source: Beilstein J Org Chem. 2011 Feb 14;7:204–9. doi: 10.3762/bjoc.7.27 (PMC3063077; doi:10.3762/bjoc.7.27)
Supplement: File 1 — Additional 2D NMR ROESY and 1H NMR data. [file Beilstein_J_Org_Chem-07-204-s001.pdf]

# Supporting information

**for**

# Chiral recognition of macromolecules with cyclodextrins: pH- and thermosensitive copolymers from *N*-isopropylacrylamide and *N*-acryloyl-D/L-phenylalanine and their inclusion complexes with cyclodextrins

Sabrina Gingter, Ella Bezdushna and Helmut Ritter*

Address: Institute of Organic Chemistry and Macromolecular Chemistry, Heinrich-Heine-University Düsseldorf, Universitätsstraße 1, D-40225 Düsseldorf, Germany

E-mail: Helmut Ritter - h.ritter@uni-duesseldorf.de

* Corresponding author

## Additional 2D NMR ROESY and $^1$H NMR data

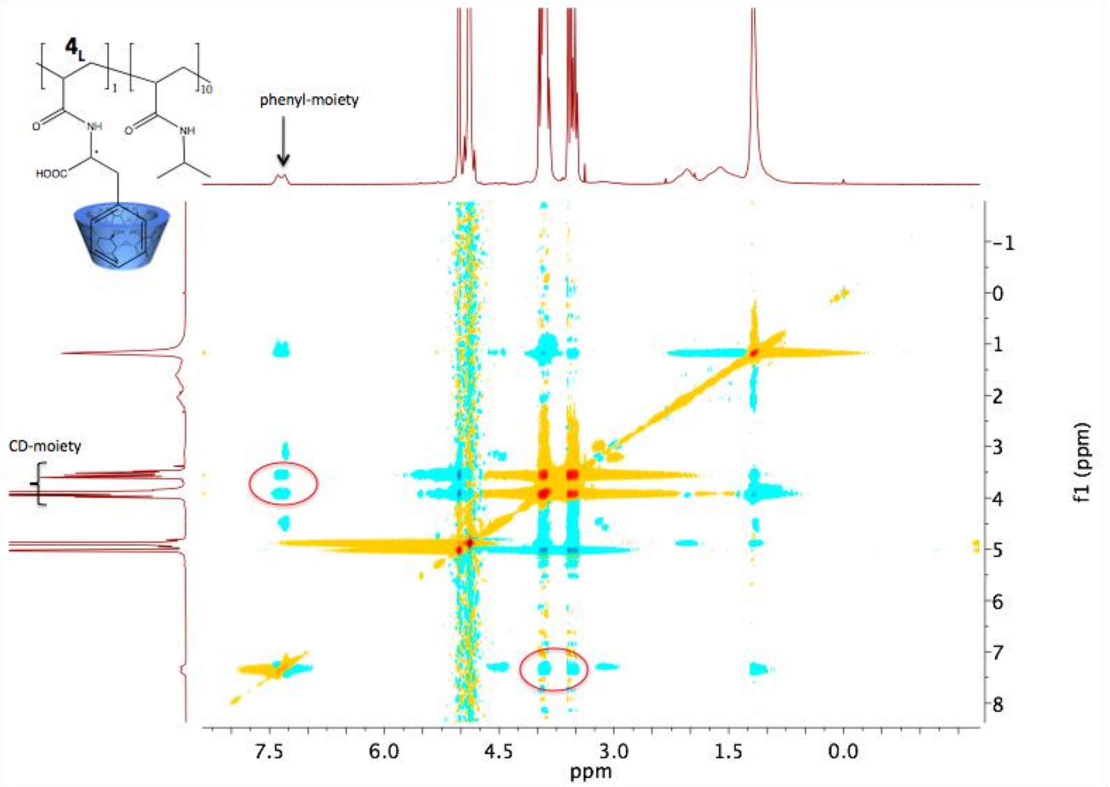


**Figure 1:** 2D NMR ROESY experiment showing the correlation between protons of the phenyl moiety of $\mathbf{4_L}$ with the β-CD protons.

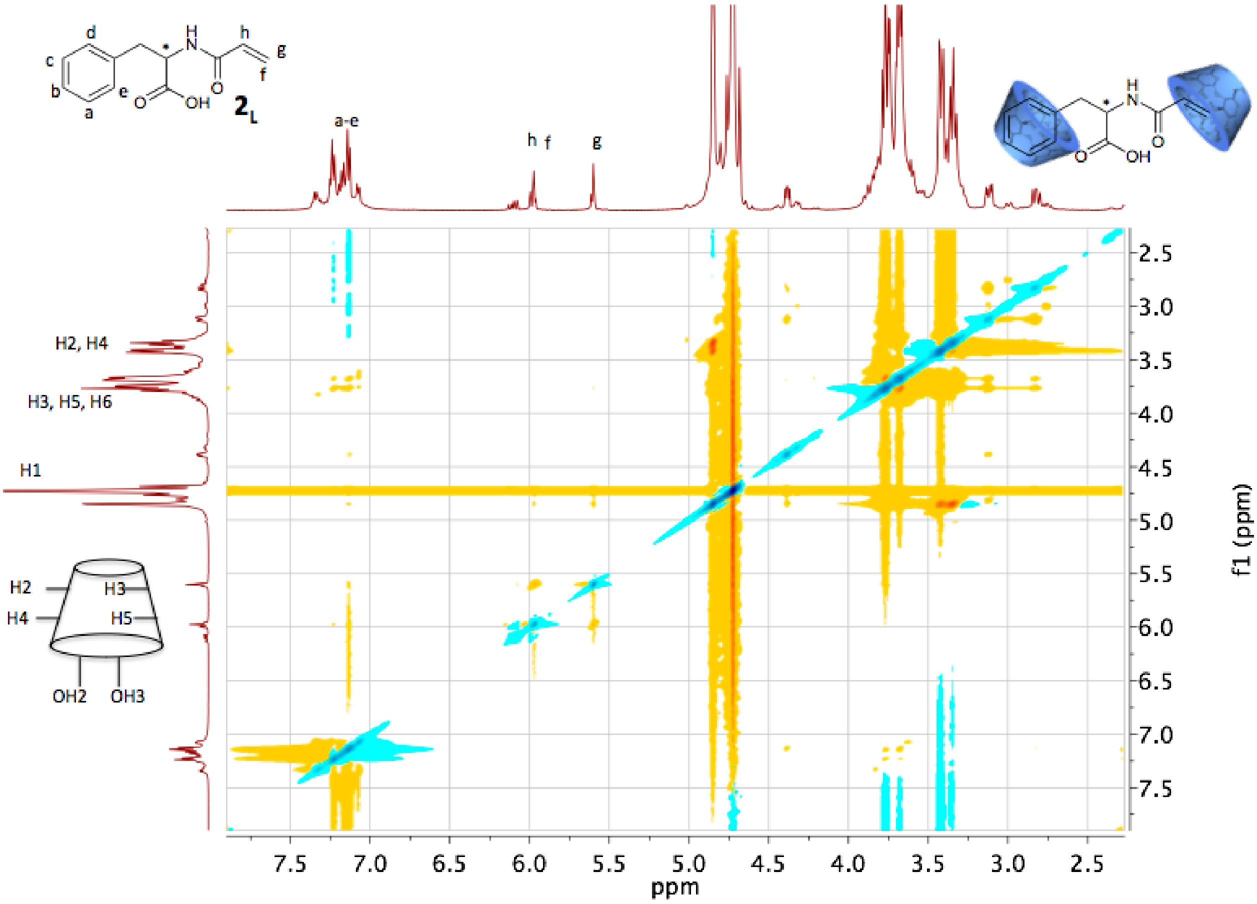


**Figure 2:** 2D NMR ROESY experiment showing the correlation between protons of the phenyl moiety of $\mathbf{2_L}$ with the β-CD protons.

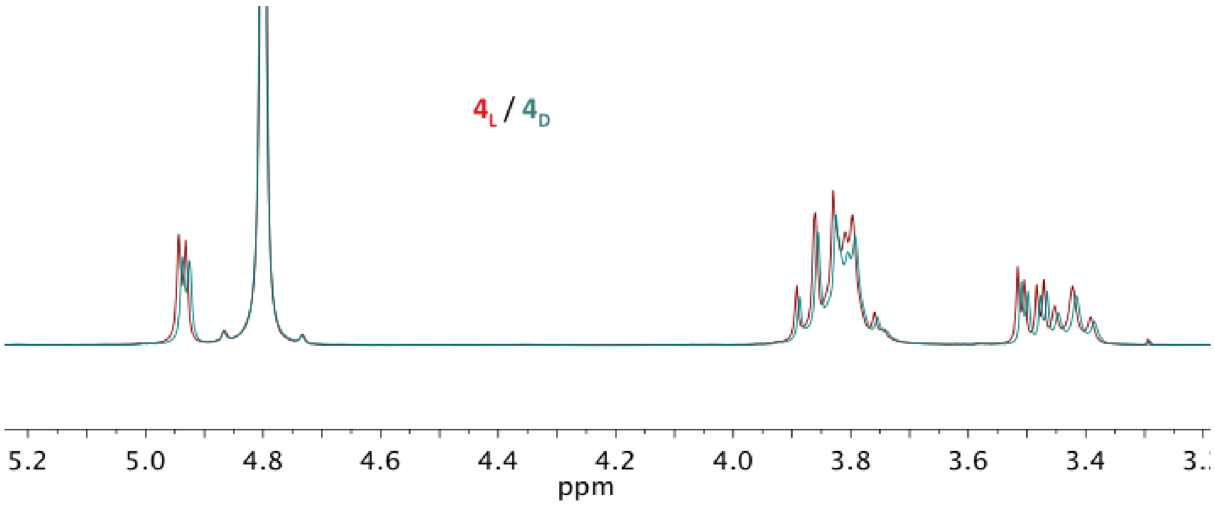


**Figure 3:** Section of $^{1}$H NMR spectra showing β-CD protons of supramolecular structures $\mathbf{4_D}$ and $\mathbf{4_L}$ in comparison exhibiting a small shift difference due to complexation with β-CD.

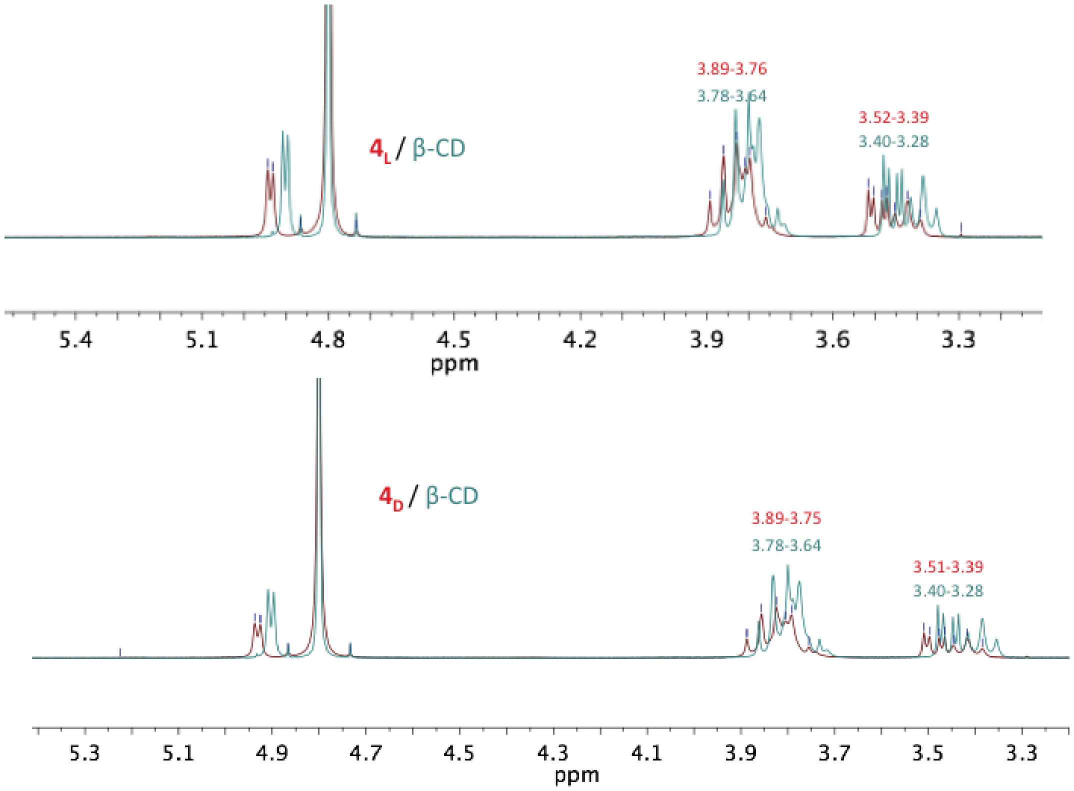


**Figure 4:** Section of $^{1}$H NMR spectra showing CD protons of supramolecular structures $\mathbf{4_D}$ and $\mathbf{4_L}$ in comparison with native β-CD exhibiting clear shift difference due to complexation with β-CD.
